# The enhanced susceptibility of ADAM-17 hypomorphic mice to DSS-induced colitis is not ameliorated by loss of RIPK3, revealing an unexpected function of ADAM-17 in necroptosis

**DOI:** 10.18632/oncotarget.24410

**Published:** 2018-02-05

**Authors:** Johaiber Fuchslocher Chico, Maren Falk-Paulsen, Anne Luzius, Carina Saggau, Barbara Ruder, Julia Bolik, Dirk Schmidt-Arras, Andreas Linkermann, Christoph Becker, Philip Rosenstiel, Stefan Rose-John, Dieter Adam

**Affiliations:** ^1^ Institut für Immunologie, Christian-Albrechts-Universität zu Kiel, 24105 Kiel, Germany; ^2^ Institut für Klinische Molekularbiologie, Christian-Albrechts-Universität zu Kiel, 24105 Kiel, Germany; ^3^ Medizinische Klinik 1, Friedrich-Alexander-Universität Erlangen-Nürnberg, 91052 Erlangen, Germany; ^4^ Institut für Biochemie, Christian-Albrechts-Universität zu Kiel, 24118 Kiel, Germany; ^5^ Medizinische Klinik und Poliklinik III, Universitätsklinikum Carl Gustav Carus, 01307 Dresden, Germany

**Keywords:** ADAM17, DSS, colitis, RIPK3, necroptosis

## Abstract

The disintegrin metalloprotease ADAM17 has a critical role in intestinal inflammation and regeneration in mice, as illustrated by the dramatically increased susceptibility of ADAM17 hypomorphic (ADAM17^ex/ex^) mice to dextran sulfate sodium (DSS)-induced colitis. Similarly, necroptosis has been implicated in inflammatory responses in the intestine. In this study, we have investigated the contribution of necroptosis to ADAM17-regulated intestinal inflammation *in vivo* by crossing ADAM17^ex/ex^ mice with mice that lack the necroptotic core protein RIPK3. Despite the loss of RIPK3, ADAM17^ex/ex^/RIPK3^−/−^ mice showed the same increased susceptibility as ADAM17^ex/ex^ mice in both acute and chronic models of DSS-induced colitis. Mice of both genotypes revealed comparable results with regard to weight loss, disease activity index and colitis-associated changes of inner organs. Histopathological analyses confirmed similar tissue destruction, loss of barrier integrity, immune cell infiltration, and cell death; serum analyses revealed similar levels of the pro-inflammatory cytokine KC. Resolving these unexpected findings, ADAM17^ex/ex^ mice did not show phosphorylation of RIPK3 and its necroptotic interaction partner MLKL during DSS-induced colitis, although both proteins were clearly expressed. Consistent with these findings, murine embryonic fibroblasts derived from ADAM17^ex/ex^ mice were protected from tumor necrosis factor (TNF)-induced necroptosis and failed to show phosphorylation of MLKL and RIPK3 after induction of necroptosis by TNF, revealing a novel, undescribed role of the protease ADAM17 in necroptosis.

## INTRODUCTION

The transmembrane protease A Disintegrin And Metalloproteinase (ADAM)17 was originally cloned as a tumor necrosis factor (TNF)-α-converting enzyme (TACE) [1, 2]. Since then, more than 80 substrates have been described that are cleaved by ADAM17. These substrates encompass adhesion molecules as well as cytokines, growth factors and their receptors [3–5]. As prominent examples, the TNF receptors TNF-R1 and TNF-R2, the interleukin-6 receptor α, L-selectin and amyloid precursor protein have been identified as ADAM17 substrates [6, 7]. By releasing the epidermal growth factor receptor (EGFR) ligands amphiregulin, epiregulin, transforming growth factor-α and heparin-binding EGF [7], ADAM17 critically contributes to EGFR-activation and thus controls vital processes such as cell growth and survival [8]. Increased expression or activity of ADAM17 has been linked to inflammatory processes as well as to the emergence of cancer [9]. Deletion of ADAM17 in mice leads to embryonic lethality caused by massive developmental defects that result from disrupted EGFR signaling [10]. Although not lethal in humans, patients suffering from ADAM17 deficiency show severe inflammatory skin and bowel disease, underscoring the important role of ADAM17 for epithelial cell homeostasis [11, 12].

To circumvent the lethality of the complete ADAM17 knockout, we have previously generated hypomorphic ADAM17^ex/ex^ mice that express only ~5% of the original levels of ADAM17 in all tissues [13]. These mice are viable, show reduced shedding of ADAM17 substrates and display tissue defects comparable to those of ADAM17-deficient mice, although less pronounced [10, 13]. Using this mouse model, we have previously shown that ADAM17 has an important function in regulating the regeneration of the intestinal epithelium. In contrast to ADAM17 wildtype (WT) mice, ADAM17^ex/ex^ mice show a dramatically increased susceptibility to dextran sulfate sodium (DSS)-induced colitis. This hypersensitivity results from impaired shedding of EGFR ligands and, in consequence, in strongly reduced regeneration of epithelial cells of the basal crypts and breakdown of the intestinal barrier [13].

Independently, studies in mouse models have implicated necroptosis of epithelial cells as a mechanism that contributes to intestinal inflammation [14–16]. In humans, necroptosis has been suggested as a crucial event that amplifies inflammation in the pathogenesis of inflammatory bowel disease [17]. Briefly, necroptosis is a caspase-independent, non-apoptotic form of regulated cell death which can be triggered by multiple stimuli such as death receptors, Toll-like receptors (TLRs), interferons, the T cell receptor, or intracellular sensors for DNA or RNA. Necroptosis depends on the activation of two core molecules, RIPK3 (receptor interacting serine-threonine protein kinase 3) and MLKL (mixed-lineage kinase domain-like). The interaction of RIPK3 with upstream activators such as the TNF-R1-associating kinase RIPK1, the TLR3/TLR4 adaptor protein TRIF or the DNA receptor DAI leads to oligomerization of RIPK3 into amyloid-like structures, its phosphorylation and subsequent activation. Active RIPK3 then interacts with and phosphorylates MLKL. This allows MLKL to oligomerize, translocate and insert into plasma membranes where it induces the collapse of membrane integrity and the necroptotic burst of the cell [18–20].

In this study, we have analyzed the contribution of necroptosis to the enhanced susceptibility of ADAM17^ex/ex^ mice to intestinal damage in DSS-induced colitis, and in particular whether this susceptibility can be ameliorated by deletion of the necroptotic core protein RIPK3. For this purpose, we have generated ADAM17^ex/ex^ mice that lack RIPK3. In contrast to our initial expectations, ADAM17^ex/ex^/RIPK3^−/−^ mice showed the same increased susceptibility as ADAM17^ex/ex^ mice in acute or chronic models of DSS-induced colitis. As the underlying mechanism, our data reveal that necroptosis is compromised in ADAM17^ex/ex^ mice even in the presence of RIPK3, and point to an unexpected role of the metalloproteinase ADAM17 as a novel regulator of necroptosis.

## RESULTS

### Comparison of ADAM17^ex/ex^ and ADAM17^ex/ex^/RIPK3^−/−^ mice in the acute colitis model

**Figure 1:**
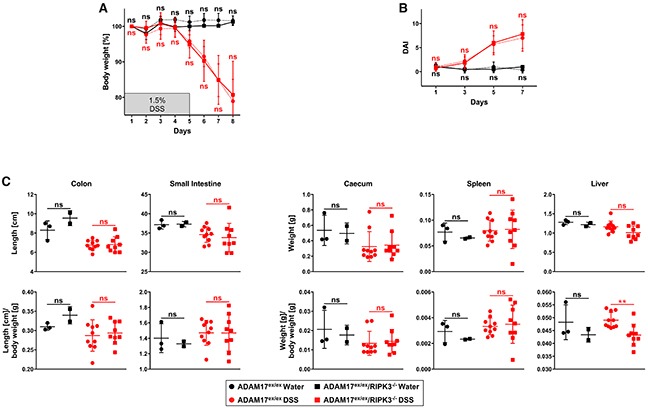
In ADAM17 hypomorphic mice, loss of RIPK3 does not ameliorate loss of body weight, disease activity index, and changes of inner organs caused by acute DSS-induced colitis **(A)** changes in body weight (relative to the starting weight on day 1) of mice treated with regular drinking water (n=3 for ADAM17^ex/ex^ and n=3 for ADAM17^ex/ex^/RIPK3^−/−^ mice) or with drinking water containing 1.5% DSS for 5 days, followed by regular drinking water (n=10 for ADAM17^ex/ex^ and n=10 for ADAM17^ex/ex^/RIPK3^−/−^ mice). **(B)** parallel calculation of the disease activity index. For A and B, data are shown as mean values ± SD. **(C)** post-mortem measurements of colon and small intestine length as well as weight of caecum, spleen and liver, shown as absolute values (upper panels) or as body weight ratios (lower panels). The horizontal bars represent the mean, error bars the SD. Differences between ADAM17^ex/ex^ and ADAM17^ex/ex^/RIPK3^−/−^ mice that do not reach significance (two-tailed unpaired Student’s *t*-test) are indicated by “ns”. ^**^, p<0.01.

We initially hypothesized that inhibition of necroptosis should ameliorate the hypersensitive response of ADAM17^ex/ex^ mice to DSS-induced colitis by preventing the necroptotic destruction of intestinal epithelial cells, and thus by reducing the ensuing inflammatory damage. To test this assumption, we crossed ADAM17^ex/ex^ mice with RIPK3-deficient mice that are unable to elicit necroptotic responses [21, 22]. We first compared the resulting ADAM17^ex/ex^/RIPK3^−/−^ mice to ADAM17^ex/ex^ animals in a model of DSS-induced acute colitis. DSS is a chemical irritant that induces intestinal epithelial cell injury, and the resulting inflammation in mice resembles the clinical and histological features of inflammatory bowel disease in humans [23]. Considering the increased susceptibility of ADAM17^ex/ex^ mice [13], we employed a very mild regimen, treating the mice with 1.5% DSS in their drinking water for 5 days, followed by a recovery period with normal drinking water for another 5 days. Despite this mild treatment, the experiment had to be terminated prematurely on day 8 due to a rapid and severe body weight loss of the ADAM17^ex/ex^ mice (Figure 1A). Other than expected, ADAM17^ex/ex^/RIPK3^−/−^ mice showed the same pronounced body weight loss (Figure 1A), and two animals died on day 8. Statistical analysis of body weight loss revealed no significant differences between ADAM17^ex/ex^ and ADAM17^ex/ex^/RIPK3^−/−^ mice. This result was unanticipated, as deletion of RIPK3 should have prevented necroptotic epithelial damage and weight loss during the toxic treatment phase with DSS, even in a prematurely terminated experiment with a reduced recovery period.

We additionally evaluated the disease activity index (DAI) of ADAM17^ex/ex^ and ADAM17^ex/ex^/RIPK3^−/−^ mice, a combined clinical score of weight loss, rectal bleeding, and diarrhea [24]. As shown in Figure 1B, the disease activity scores of both mouse models were comparable over the course of acute colitis and did not show statistically significant differences.

During colitis, edema is increased and colon length is shortened [25]. We therefore assessed differences in colon length as an indicator of the severity of injury. Since DSS-induced damage can also extend to the small intestine [26], we additionally monitored for differences in the length of the small intestine. We also determined the weights of the caecums to evaluate colitis-associated caecum shrinkage and recorded spleen and liver weights as systemic parameters of inflammation [27, 28]. As shown in Figure 1C (upper panels), DSS-treated ADAM17^ex/ex^ and ADAM17^ex/ex^/RIPK3^−/−^ mice exhibited comparable changes in the absolute length of colons and small intestines as well as the weight of caecums, spleens and livers. This pattern persisted when we recalculated these parameters as body weight ratios (Figure 1C, lower panels). Statistical analysis did not reveal significant differences between ADAM17^ex/ex^ and ADAM17^ex/ex^/RIPK3^−/−^ animals, except for the liver weight/body weight ratio in ADAM17^ex/ex^/RIPK3^−/−^ mice, which was slightly decreased compared to ADAM17^ex/ex^ animals (p=0.0032).

### Histological evaluation of acute colitis in ADAM17^ex/ex^ and ADAM17^ex/ex^/RIPK3^−/−^ mice

**Figure 2:**
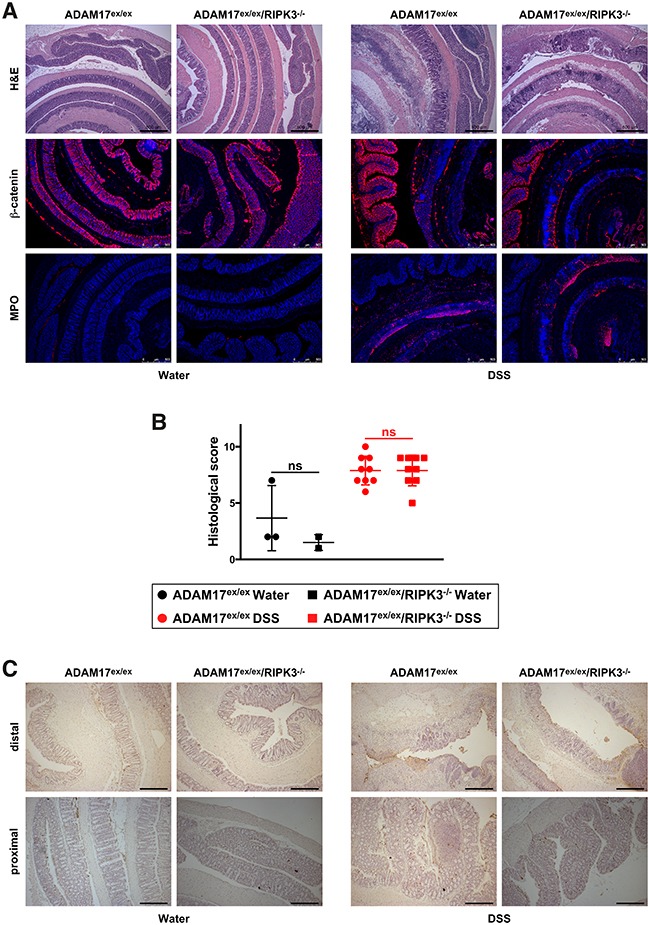
DSS-induced acute colitis causes similar tissue destruction, loss of barrier integrity, immune cell infiltration, and cell death in ADAM17^ex/ex^ and ADAM17^ex/ex^/RIPK3^−/−^ mice **(A)** colon sections from ADAM17^ex/ex^ and ADAM17^ex/ex^/RIPK3^−/−^ control mice (water) or from animals undergoing acute colitis (DSS) stained with H&E, or with antibodies for β-catenin or myeloperoxidase (MPO). Bars, 500 μm. **(B)** combined histological score of inflammatory cell infiltration, tissue damage and ulceration. The horizontal bars represent the mean, error bars the SD. Analysis by two-tailed unpaired Student’s *t*-test revealed no significant differences between ADAM17^ex/ex^ and ADAM17^ex/ex^/RIPK3^−/−^ mice within the water and DSS groups (indicated by “ns”). **(C)** representative pictures of TUNEL staining (cell death). Both distal (upper panels) and proximal colon sections (lower panels) are shown. In the distal colon, large-scale epithelial loss caused by DSS is more prominently visible, but impedes the detection of TUNEL-positive dead cells (dark spots), which are more clearly detectable in the proximal colon sections.

Histological examination of fixed tissue from colon biopsies showed extensive infiltration of leukocytes into the lamina propria, loss of crypt structure, epithelial cell loss and tissue destruction in hematoxylin and eosin (H&E)-stained sections of DSS-treated mice compared to the water controls. Likewise, staining for the epithelial cell marker β-catenin revealed a pronounced loss of barrier integrity, and detection of the inflammation marker myeloperoxidase demonstrated a massive neutrophil/monocyte infiltration in DSS-treated mice (Figure 2A). Nevertheless, and consistent with our above results, these effects were equally evident in ADAM17^ex/ex^ and ADAM17^ex/ex^/RIPK3^−/−^ mice. In the water-treated control groups, histological signs of inflammation were not detected (Figure 2A). After scoring in a blinded fashion for inflammatory cell infiltration, tissue damage and ulceration, the associated histology scores confirmed similar patterns of disease severity in ADAM17^ex/ex^ and ADAM17^ex/ex^/RIPK3^−/−^ mice (Figure 2B). Furthermore, histological analysis showed no obvious differences in cell death (TdT-mediated dUTP nick end labeling (TUNEL) staining) between mice of both genotypes (Figure 2C). To follow up on the differences in the liver weight/body weight ratios that we had observed in Figure 1C, we additionally analyzed H&E-stained liver sections of ADAM17^ex/ex^ and ADAM17^ex/ex^/RIPK3^−/−^ mice but did not detect any dissimilarities in disease severity at the histological level (data not shown).

### KC serum levels in ADAM17^ex/ex^ and ADAM17^ex/ex^/RIPK3^−/−^ mice undergoing acute colitis

**Figure 3:**
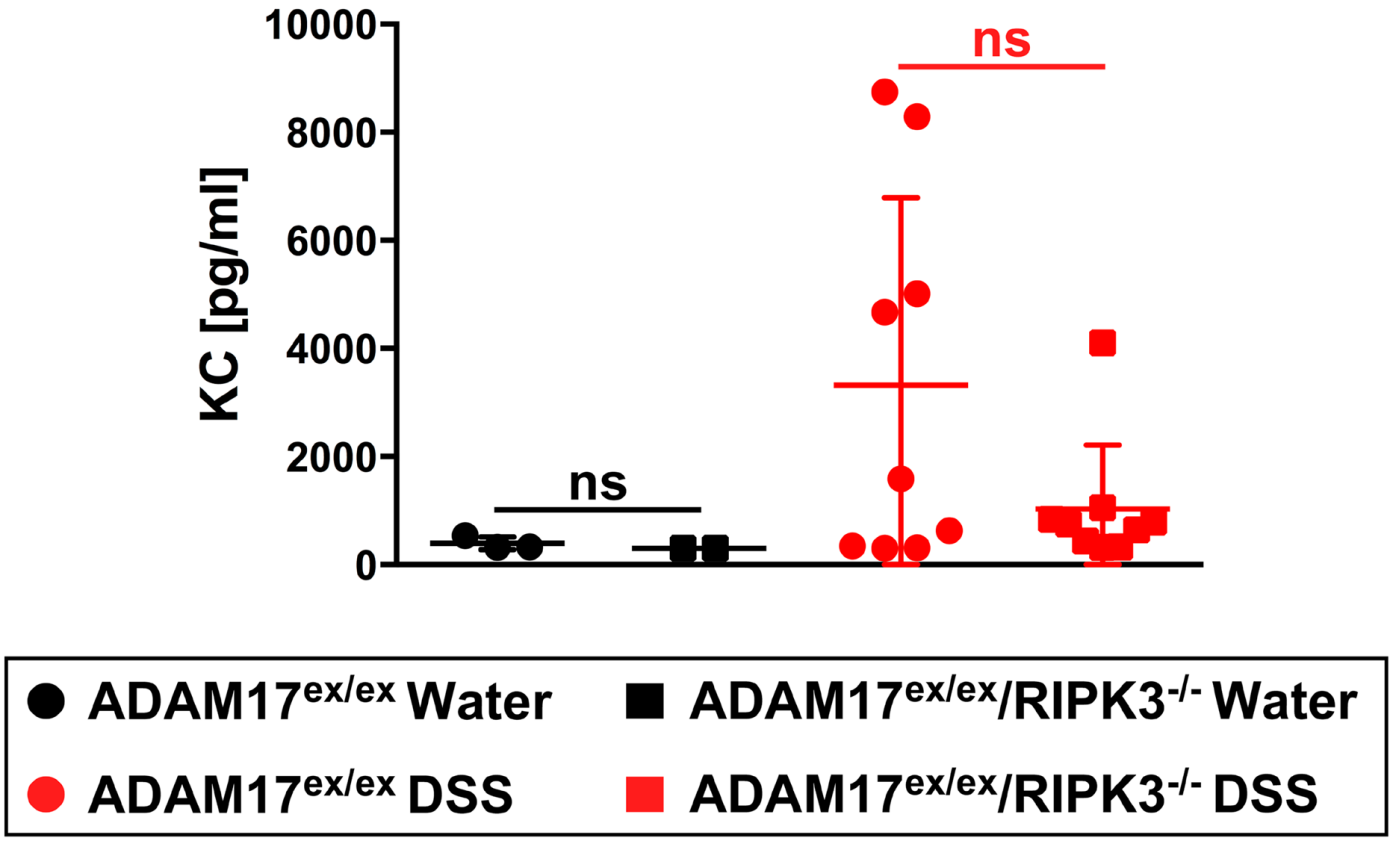
Impact of acute colitis on KC serum levels in ADAM17^ex/ex^ and ADAM17^ex/ex^/RIPK3^−/−^ mice Levels of the pro-inflammatory cytokine KC were quantified from the serum of ADAM17^ex/ex^ and ADAM17^ex/ex^/RIPK3^−/−^ control mice (water) or undergoing acute colitis (DSS). Horizontal bars represent the mean, error bars the SD. Analysis by two-tailed unpaired Student’s *t*-test revealed no differences with statistical significance within the respective water- or DSS-treated groups (indicated by “ns”).

The pro-inflammatory cytokine KC (keratinocyte-derived cytokine, the murine homologue of human IL-8 [29]) mediates neutrophil infiltration and its expression is upregulated in DSS-induced colitis [30]. Conversely, genetic deletion of KC protects mice against DSS-induced colitis [31]. When we analyzed KC serum levels, more ADAM17^ex/ex^ than ADAM17^ex/ex^/RIPK3^−/−^ mice showed an elevation in the DSS-treated group. However, these differences did not reach statistical significance (Figure 3).

### Induction of chronic colitis in ADAM17^ex/ex^ and ADAM17^ex/ex^/RIPK3^−/−^ mice

**Figure 4:**
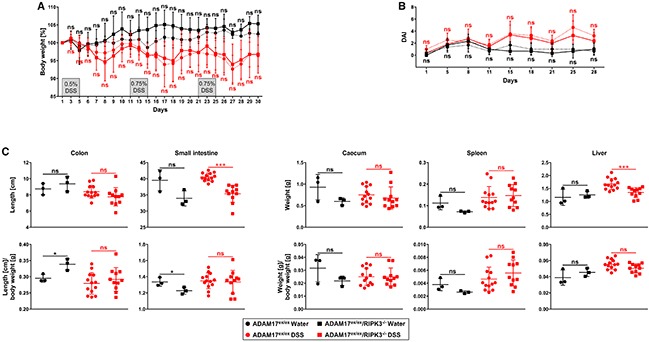
Deletion of RIPK3 does not affect loss of body weight, disease activity index, and changes of inner organs caused by chronic DSS-induced colitis in ADAM17 hypomorphic mice **(A)** changes in body weight are shown relative to the initial weight on day 1 for mice supplied with regular drinking water (n=3 for ADAM17^ex/ex^ and n=3 for ADAM17^ex/ex^/RIPK3^−/−^ mice) or for mice given cycles of DSS/regular drinking water for 5 days each (total of 3 cycles; 0.5% DSS in cycle 1, 0.75% DSS in cycles 2 and 3; n=15 for ADAM17^ex/ex^ and n=13 for ADAM17^ex/ex^/RIPK3^−/−^ mice; two animals from each group had to be prematurely killed due to severe weight loss). **(B** and **C)** parallel analysis of the disease activity and inner organs as in Figure 1B, C. For A and B, data are shown as mean values ± SD. For C, the horizontal bars represent the mean, error bars the SD. Differences between ADAM17^ex/ex^ and ADAM17^ex/ex^/RIPK3^−/−^ mice that do not reach significance (two-tailed unpaired Student’s *t*-test) are indicated by “ns”. ^*^, p<0.05; ^***^, p<0.001.

Given the pronounced response that we had seen in ADAM17^ex/ex^ and ADAM17^ex/ex^/RIPK3^−/−^ mice in the acute colitis model, we speculated that the employed treatment regimen of 1.5% DSS might have overpowered any protection that lack of RIPK3 might have conferred to the ADAM17^ex/ex^/RIPK3^−/−^ mice. Moreover, in acute and chronic inflammatory diseases, the underlying pathophysiologic principles are not identical. Therefore, a valid statement about the impact of RIPK3 on the inflammatory process can only be made after its analysis in both acute and chronic models. We thus decided to reanalyze ADAM17^ex/ex^ and ADAM17^ex/ex^/RIPK3^−/−^ mice in a chronic colitis model, and to further reduce the DSS concentration to 0.5%. Mice were treated with DSS for 5 days, followed by a regeneration phase of 5 more days with regular drinking water for a total of three cycles, i.e., 30 days. Since 0.5% DSS caused only insignificant weight loss during the first five days of treatment (Figure 4A), we increased the DSS concentration to 0.75% in the second and third cycle. Relative to control animals supplied with regular drinking water, treatment with DSS induced a significant loss of body weight (p=0.044 for ADAM17^ex/ex^ and p=0.0218 for ADAM17^ex/ex^/RIPK3^−/−^ mice on day 30). However, in ADAM17^ex/ex^ vs. ADAM17^ex/ex^/RIPK3^−/−^ mice, weight loss was comparable without statistically significant differences (Figure 4A). Likewise, the DAI of ADAM17^ex/ex^ and ADAM17^ex/ex^/RIPK3^−/−^ showed a similar course over the duration of the chronic colitis experiment and no differences that were statistically significant (Figure 4B). Finally, analysis of colon and small intestine length and weight of caecum, spleen and liver revealed a similar picture as in the acute colitis model: differences of statistical significance between DSS-treated ADAM17^ex/ex^ and ADAM17^ex/ex^/RIPK3^−/−^ mice were only found in the absolute length of the small intestine (p<0.0001) and the absolute liver weight (p=0.0008), but were no longer apparent when recalculated relative to the body weight (Figure 4C).

### Histological assessment of chronic colitis in ADAM17^ex/ex^ and ADAM17^ex/ex^/RIPK3^−/−^ mice

**Figure 5:**
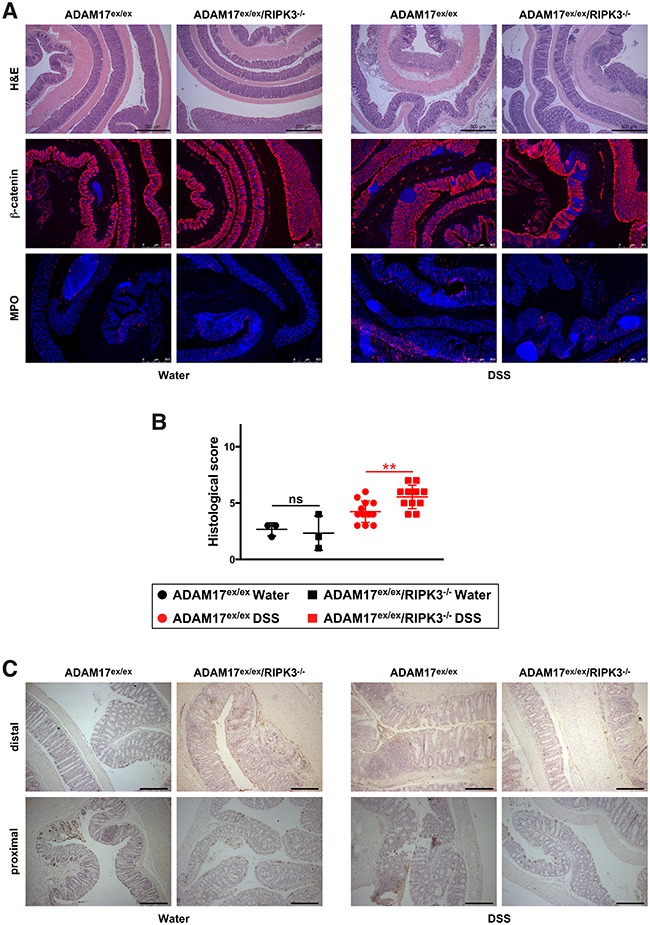
ADAM17^ex/ex^ and ADAM17^ex/ex^/RIPK3^−/−^ mice show comparable tissue destruction, loss of barrier integrity, immune cell infiltration, and cell death in DSS-induced chronic colitis **(A)** H&E, β-catenin or MPO stainings of colon sections from ADAM17^ex/ex^ and ADAM17^ex/ex^/RIPK3^−/−^ control mice (water) or animals supplied with DSS to induce chronic colitis. Bars, 500 μm. **(B)** inflammatory cell infiltration, tissue damage and ulceration were summarized in to a combined score as in Figure 2B. The horizontal bars represent the mean, error bars the SD. Non-significant statistical differences between ADAM17^ex/ex^ and ADAM17^ex/ex^/RIPK3^−/−^ mice (two-tailed unpaired Student’s *t*-test) are indicated by “ns”. ^**^, p<0.01. **(C)** analysis of cell death by TUNEL staining (cell death) as in Figure 2C. Both distal (upper panels) and proximal colon sections (lower panels) are shown. Other than in the acute colitis model, epithelial loss in the distal colon is less pronounced, but uniform between ADAM17^ex/ex^ and ADAM17^ex/ex^/RIPK3^−/−^ mice.

As in the acute colitis model, histological analyses demonstrated loss of crypt structure, epithelial cell loss and tissue destruction, loss of barrier integrity, increased neutrophil infiltration and increased cell death in colon sections from DSS-treated mice, showing similar severity in ADAM17^ex/ex^ and ADAM17^ex/ex^/RIPK3^−/−^ mice (Figure 5A, 5C). In line with the less aggressive chronic colitis model, blinded scoring for inflammatory cell infiltration, tissue damage and ulceration revealed an overall score in DSS-treated mice (Figure 5B) that was reduced in comparison to acute colitis (Figure 2B). Although scoring showed a statistically significant difference between DSS-treated ADAM17^ex/ex^ and ADAM17^ex/ex^/RIPK3^−/−^ mice (p=0.0037), the means of the scores differed only marginally by 1.3 points, with the ADAM17^ex/ex^/RIPK3^−/−^ mice showing more, but not less damage, inconsistent with an increased protection in ADAM17^ex/ex^/RIPK3^−/−^ mice. As in the acute colitis model, TUNEL staining revealed no obvious differences in cell death between mice of both genotypes (Figure 5C).

### Impact of chronic colitis on KC levels in ADAM17^ex/ex^ and ADAM17^ex/ex^/RIPK3^−/−^ mice

**Figure 6:**
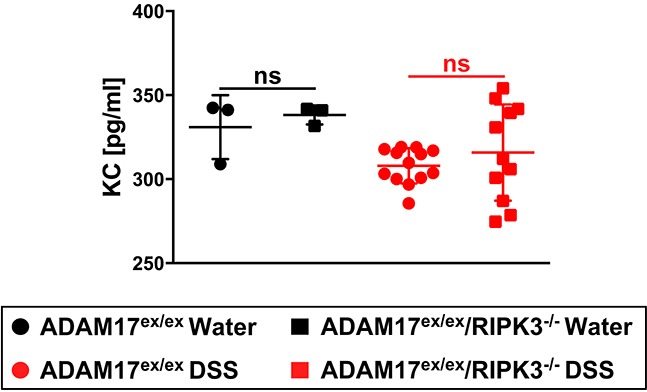
Effect of chronic colitis on KC serum levels in ADAM17^ex/ex^ and ADAM17^ex/ex^/RIPK3^−/−^ mice KC levels were quantified from the serum of ADAM17^ex/ex^ and ADAM17^ex/ex^/RIPK3^−/−^ control mice (water) or undergoing chronic colitis (DSS). Horizontal bars represent the mean, error bars the SD. Analysis by two-tailed unpaired Student’s *t*-test revealed no statistically significant differences within the respective water- or DSS-treated groups (indicated by “ns”).

In analyses for the pro-inflammatory cytokine KC, ADAM17^ex/ex^ and ADAM17^ex/ex^/RIPK3^−/−^ mice exhibited comparable KC serum levels following challenge with DSS, without statistically significant differences (Figure 6).

In summary, the combined data from the above acute and chronic colitis experiments show that the course and severity of disease in each model are very similar, if not identical in ADAM17^ex/ex^ and ADAM17^ex/ex^/RIPK3^−/−^ mice, and strongly support the assumption that the functions of RIPK3 are dispensable for DSS-induced colitis in ADAM17 hypomorphic mice.

### DSS-induced colitis does not trigger necroptosis in ADAM17^ex/ex^ mice

**Figure 7:**
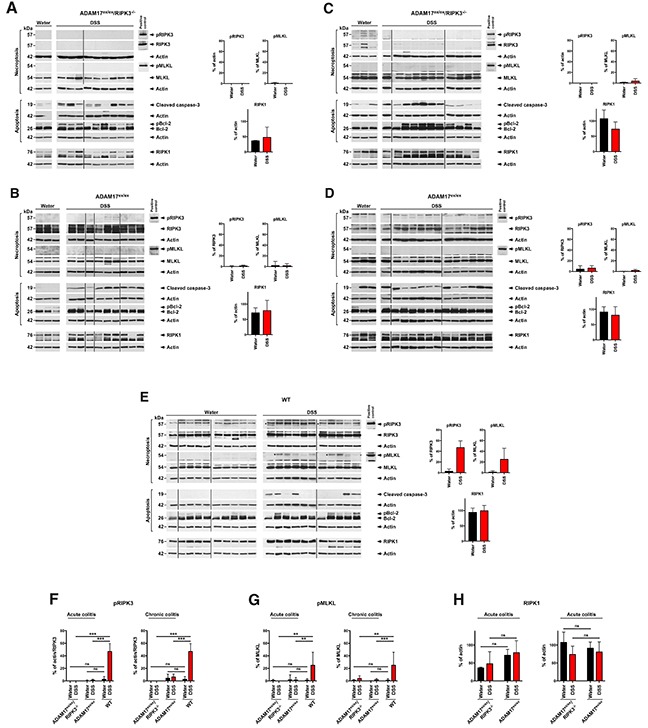
DSS-induced colitis does not induce necroptosis in the colons of ADAM17^ex/ex^ mice **(A)** colon samples from ADAM17^ex/ex^/RIPK3^−/−^ control mice supplied with regular drinking water (water) or with DSS to induce acute colitis (DSS) were analyzed by Western blot for markers of necroptosis (pRIPK3, pMLKL) or apoptosis (cleaved caspase-3, pBcl-2 (detected with Bcl-2 antibody as a form of Bcl-2 with higher molecular weight [32])), as well as for expression of RIPK1. Detection of actin served as control for equal loading. Quantifications for pRIPK3, pMLKL and RIPK1 are shown to the right. To increase the clarity of presentation, positive control lysates are depicted separately, and the panels are shown cropped and re-ordered. The uncropped Western blots in their original order with all positive controls are shown in Supplementary Figure 1. **(B)** parallel analysis for acute colitis in ADAM17^ex/ex^ mice. The pRIPK3 and pMLKL panels are deliberately overexposed to show that even at longer exposure times, no specific signal is detectable. **(C)** colon samples from ADAM17^ex/ex^/RIPK3^−/−^ or **(D)** ADAM17^ex/ex^ mice treated with water or DSS to induce chronic colitis were analyzed as in A, B. **(E)** Parallel analysis for colon samples from DSS-treated WT mice. Asterisks indicate bands which correspond to the expected size of pRIPK3 and pMLKL. **(F)** statistical analysis of pRIPK3, **(G)** pMLKL, or **(H)** RIPK1 expression. The horizontal bars represent the mean, error bars the SD. ns, not significant, ^**^, p<0.01, ^***^, p<0.001 (two-tailed unpaired Student’s *t*-test).

To obtain further insight why loss of RIPK3 had not improved the response of ADAM17^ex/ex^ mice to DSS-induced acute and chronic colitis, we performed Western blots for molecular markers of cell death. In colon samples from ADAM17^ex/ex^/RIPK3^−/−^ mice, no signal for phosphorylated or unphosphorylated RIPK3 was detectable, consistent with the RIPK3 knockout in these mice (Figure 7A). Also, MLKL was detectable only in its inactive, unphosphorylated form, confirming the absence of necroptosis in the colons of ADAM17^ex/ex^/RIPK3^−/−^ mice. In contrast, cleaved, active caspase-3 as a marker for apoptotic cell death was elevated in samples from DSS- vs. water-treated control mice. A similar pattern emerged when we analyzed phosphorylation of Bcl-2 (pBcl-2) as a second marker for apoptosis [32], suggesting that the tissue damage and TUNEL-positive signals seen above in ADAM17^ex/ex^/RIPK3^−/−^ mice were mainly caused by apoptosis. Parallel Western blots from colon samples from ADAM17^ex/ex^ mice confirmed the presence of both RIPK3 and MLKL as anticipated (Figure 7B). Surprisingly, however, even in the longer exposures shown in Figure 7B, we did not detect signals specific for phosphorylated RIPK3 (pRIPK3) or phosphorylated MLKL (pMLKL), suggesting that in ADAM17^ex/ex^ mice, induction of acute colitis by DSS does not result in necroptotic signaling despite the presence of RIPK3 and MLKL. Although pBcl-2 was not detectable for unknown reasons in ADAM17^ex/ex^/RIPK3^−/−^ mice, cleaved caspase-3 displayed an increased signal in samples from DSS-treated ADAM17^ex/ex^ mice, showing that unlike necroptosis, apoptosis *is* induced in ADAM17^ex/ex^ mice in the course of acute colitis (Figure 7B).

Next, we included colon samples from the chronic colitis model in our analyses. Identical to acute colitis, colon samples from ADAM17^ex/ex^/RIPK3^−/−^ mice lacked signals specific for RIPK3 and pRIPK3 (as expected), and no signal specific for phosphorylated MLKL was detectable despite the presence of MLKL in all samples (Figure 7C). As seen in the acute colitis model, DSS treatment also increased the signals for cleaved caspase-3 and pBcl-2 (Figure 7C). In ADAM17^ex/ex^ mice subjected to chronic colitis, we again detected no signals specific for pRIPK3 and pMLKL despite the clear presence of inactive, unphosphorylated RIPK3 and MLKL in all samples (Figure 7D), further supporting our assumption that necroptotic signaling is compromised in ADAM17^ex/ex^ mice. As in the acute colitis model, pBcl-2 was not detectable in samples from DSS-treated mice. However, the results for cleaved caspase-3 were again consistent (except for two mice from the water control group that showed cleavage of caspase-3 for unknown reasons) with those obtained in ADAM17^ex/ex^/RIPK3^−/−^ mice or in the acute colitis models (i.e., apoptosis was induced, Figure 7D).

As an additional control to determine whether the results seen in ADAM17^ex/ex^ and in ADAM17^ex/ex^/RIPK3^−/−^ mice were indeed due to a compromised necroptotic response, we analyzed colon samples from water- or DSS-treated WT mice generated in an independent study. As expected, RIPK3 and MLKL were expressed in all samples (Figure 7E). Moreover, a faint band corresponding to the expected size of pRIPK3 was detectable in DSS-treated samples (Figure 7E, asterisks, in addition to several nonspecific bands), but absent in the water controls. Likewise, pMLKL was detectable in the majority of the colon samples from DSS-treated mice (but absent or visible only at very low levels in the water controls, Figure 7E), in summary indicating that in WT mice, treatment with DSS *does* induce necroptotic signaling. For cleaved caspase-3 (apoptotic signaling), we obtained comparable results as in ADAM17^ex/ex^/RIPK3^−/−^ and ADAM17^ex/ex^ mice, i.e., cleaved caspase-3 was increased in most samples from DSS-, but not water-treated mice. Similar results were obtained with pBcl-2. When we quantified the pRIPK3 and pMLKL levels from the above Western blots (Figure 7A-7E), analysis of the obtained data confirmed that the differences between the levels of pRIPK3 and pMLKL in samples from WT mice vs. ADAM17^ex/ex^ and ADAM17^ex/ex^/RIPK3^−/−^ mice were statistically significant (Figure 7F, 7G).

Noteworthy, beyond acting as an upstream activator of RIPK3 [18–20], RIPK1 can also have an inhibiting role in RIPK3-dependent necroptosis [33–35]. Therefore, we considered the possibility that RIPK1 was activated or upregulated in ADAM17^ex/ex^ mice in comparison to ADAM17^ex/ex^/RIPK3^−/−^ mice. However, an analysis of RIPK1 activation by pRIPK1 Western blot from the available mouse lysates was not possible, because, to the best of our knowledge, antibodies against active, phosphorylated RIPK1 are available only against human, but not mouse pRIPK1. Therefore, we alternatively performed Western blots for RIPK1 from all colon samples and quantified the RIPK1 levels against actin (Figure 7A-7E). However, in both acute and chronic models, differences in the expression levels of RIPK1 in ADAM17^ex/ex^ vs. ADAM17^ex/ex^/RIPK3^−/−^ mice did not reach statistical significance (Figure 7H).

**Figure 8:**
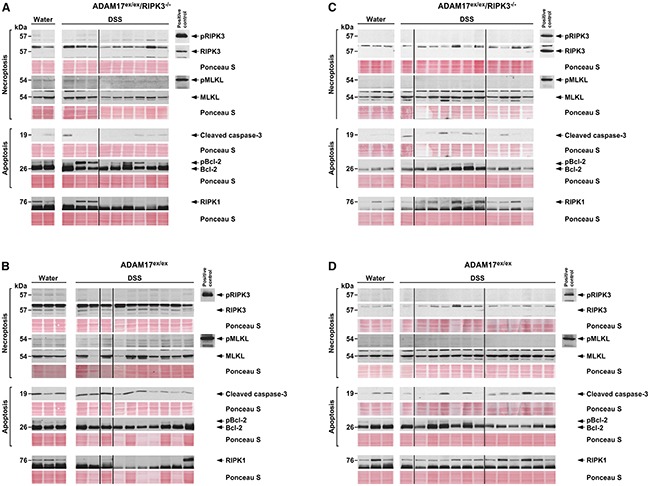
Necroptosis is absent in livers from ADAM17^ex/ex^ mice undergoing DSS-induced colitis Liver samples from ADAM17^ex/ex^/RIPK3^−/−^ **(A, C)** and ADAM17^ex/ex^ mice **(B, D)** subjected to acute (A, B) or chronic (C, D) colitis experiments were analyzed by Western blot as in Figure 7. Since actin was not detectable, or only at very low levels, equal loading was alternatively validated by staining with Ponceau S. The uncropped Western blots in their original order with all positive controls are shown in Supplementary Figure 2.

We furthermore investigated whether the compromised necroptotic response seen in ADAM17^ex/ex^ mice was a tissue-specific, colon-restricted phenomenon. We performed Western blots from liver as an independent organ that is affected systemically by DSS-induced colitis [28], using samples from both acute and chronic colitis experiments. Again, in samples from ADAM17^ex/ex^/RIPK3^−/−^ mice, RIPK3 was absent, MLKL was clearly expressed, and pRIPK3 and pMLKL were not detectable, consistent with the genotype causing defective necroptotic signaling (Figure 8A, 8C). Induction of apoptosis (i.e., cleavage of caspase-3 and phosphorylation of Bcl-2) was less prominent than in the colon samples, but still detectable (Figure 8A, 8C). In liver samples from ADAM17^ex/ex^ mice, the same pattern emerged (except for the anticipated presence of RIPK3, Figure 8B, 8D). Like in the colon samples, RIPK1 showed comparable expression levels in ADAM17^ex/ex^ and ADAM17^ex/ex^/RIPK3^−/−^ mice (Figure 8A-8D). These results suggest that necroptotic signaling in ADAM17^ex/ex^ mice is indeed compromised in the whole organism and that the defect is not restricted to the colon.

### Embryonic fibroblasts from ADAM17^ex/ex^ mice are protected from TNF-induced necroptosis

**Figure 9:**
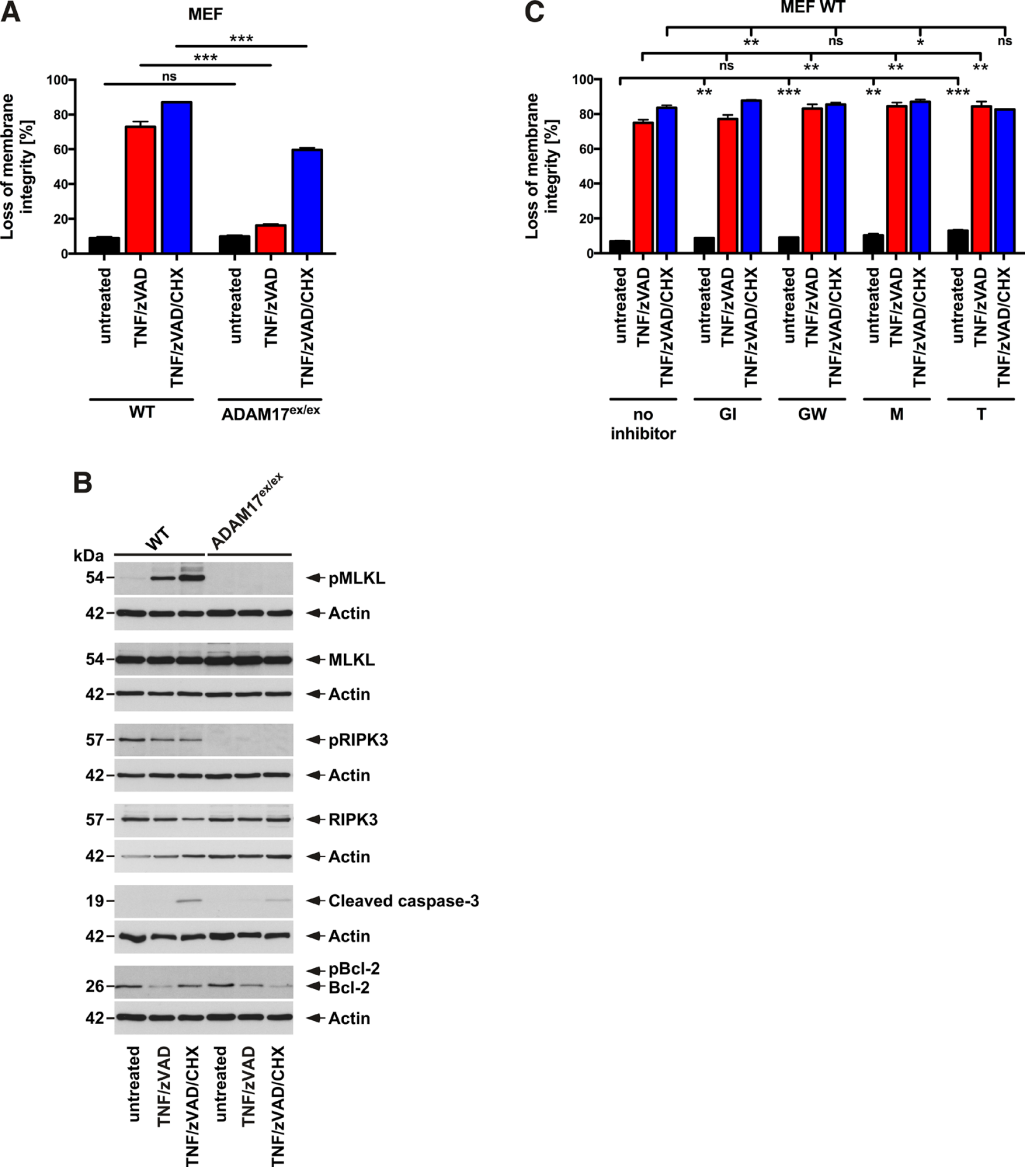
ADAM17 hypomorphic MEF are protected from TNF-induced necroptosis **(A)** MEF from WT and ADAM17^ex/ex^ mice were left untreated or preincubated for 30 min with 20 μM zVAD-fmk in the absence or presence of 1 μg/ml CHX, followed by stimulation with 100 ng/ml TNF. After 16 h, loss of membrane integrity was determined as a marker for cell death by flow cytometric analysis of PI-positive cells. Each measurement represents the mean of three parallel determinations, error bars indicate the corresponding SD. **(B)** in parallel, cell lysates were analyzed by Western blot for presence of pMLKL, MLKL, pRIPK3, RIPK3, cleaved caspase-3 and pBcl-2. Detection of actin served as a loading control. **(C)** WT MEF were left untreated or preincubated for 30 min with 3 μM GW280264X (GW), 3 μM GI254023X (GI), 10 μM marimastat (M) or 50 μM TAPI-1 (T) in combination with zVAD-fmk, CHX and subsequent addition of TNF as in A before cell death was analyzed. Each measurement represents the mean of three parallel determinations, error bars indicate the corresponding SD. ns, not significant, ^*^, p<0.05, ^**^, p<0.01, ^***^, p<0.001 (two-tailed unpaired Student’s *t*-test).

In contrast to the complex pathophysiological events that occur during DSS-induced colitis, TNF-induced necroptosis is elicited by a defined trigger and represents one of the best-studied model system for necroptosis [18–20]. Using this system, we investigated whether the protective effects of hypomorphic ADAM17 extended beyond colitis-associated necroptosis. As shown in Figure 9A, treatment of murine embryonic fibroblasts (MEF) from WT mice with TNF and the broad-spectrum caspase inhibitor benzyloxycarbonyl-Val-Ala-Asp(OMe)-fluoromethylketone (zVAD-fmk) or with TNF/zVAD in combination with cycloheximide (CHX, to increase sensitivity for necroptosis) was strongly cytotoxic. In contrast, ADAM17^ex/ex^ MEF were protected from TNF/zVAD-induced cytotoxicity, even in the presence of CHX. In Western blots shown in Figure 9B, treated WT MEF displayed strong phosphorylation of MLKL, confirming that cell death indeed occurred by necroptosis. In contrast, but fully consistent with our results from the colitis models, pMLKL was absent from treated ADAM17^ex/ex^ MEF. As a second marker for necroptosis, pRIPK3 showed the same pattern (although a signal was also detected in unstimulated WT MEF for unknown reasons). Western blots for cleaved caspase-3 and pBcl-2 confirmed the absence of apoptosis (the slight amounts of cleaved caspase-3 present in cells treated with TNF/zVAD/CHX for 16 h have also been observed by others in late stages of necroptosis and are most likely due to an unspecific proteolysis of caspases in disintegrating cells [36]).

To investigate whether the cleavage activity of ADAM17 is important for its function in necroptosis, we treated WT MEF with TNF/zVAD or TNF/zVAD/CHX in the absence or presence of GW280264X (an inhibitor of ADAM17 and ADAM10), GI254023X (an inhibitor of ADAM10, to account for effects of ADAM10 seen with GW280264X), marimastat (a broad-spectrum inhibitor of metalloproteinases) and TAPI-1 (an inhibitor of ADAM17 and other metalloproteinases). However, none of the inhibitors conferred protection from necroptosis. On the contrary, statistically significant changes caused by the inhibitors solely originated from increased, not decreased cell death (Figure 9C).

## DISCUSSION

ADAM17 is a central regulator of intestinal homeostasis, and mice hypomorphic for ADAM17 are highly susceptible to DSS-induced colitis [13]. We therefore considered them as a particularly sensitive and suitable system to study the impact of necroptosis on intestinal inflammation, also with regard to possible interconnections of RIPK3 and ADAM17 signaling. A role of necroptosis in intestinal inflammation has been suggested by studies in other mouse models [14–16], and necroptosis has also been implicated in the pathogenesis of inflammatory bowel disease in humans [17]. As independent evidence for the activation of necroptotic signaling in intestinal inflammation, we have detected signals corresponding to pRIPK3 and pMLKL in colon samples from DSS-treated WT mice in our own experiments.

Further support for the concept that necroptosis contributes to intestinal inflammation comes from a study by Liu and colleagues. The authors showed that in acute DSS-induced colitis, treatment of mice with the RIPK1 inhibitor necrostatin-1 triggered anti-inflammatory and antitumorigenic effects that attenuated intestinal inflammation and colitis-associated tumor growth in mice [37]. However, these results must be interpreted with some caution, since necrostatin-1 also nonspecifically inhibits indoleamine-2,3-dioxygenase, an enzyme that likewise participates in the modulation of inflammation via the innate and adaptive immune system [38]. In addition, although necroptosis clearly occurs in DSS-induced colitis, unequivocal evidence that necroptosis indeed participates in human inflammatory bowel disease remains limited [18]. The corresponding analyses are complicated by the fact that novel, necroptosis-independent functions have been described by which RIPK3 controls inflammatory responses [39, 40]. Accordingly, Moriwaki *et al.* have reported that in their hands, the absence of RIPK3 exacerbates rather than protects from DSS-induced colitis. They suggested that DSS-induced tissue injury occurs in a RIPK3-independent manner, and that non-necroptotic functions of RIPK3 are important for subsequent tissue repair by inducing an axis of the cytokines IL-23, IL-1β, and IL-22 in bone marrow-derived dendritic cells [41]. In direct contrast, Newton and colleagues have likewise investigated DSS-induced colitis in RIPK3-deficient mice, and found no impact of loss of RIPK3 at all, i.e., RIPK3^−/−^ and WT mice showed identical responses [42]. As discussed by Newton *et al.* and Moriwaki *et al.* [41, 42], differences in the time or dosage of DSS-treatment and commensal microbiota may be responsible for these contradicting results. However, at present, the exact roles of RIPK3 and necroptosis in intestinal inflammation remain controversial.

In our own study, we have obtained results that are consistent with those of Newton *et al.* in that we also did not see a significant effect of RIPK3 deletion on the course of DSS-induced acute and chronic colitis. However, here it is important to keep in mind that we utilized ADAM17^ex/ex^ and not WT mice for our experiments. Since our results implicate that necroptotic signaling is already compromised in ADAM17^ex/ex^ mice in the first place, it is not possible to determine whether the pro-necroptotic functions of RIPK3 are important or dispensable for the course of colitis in these mice. However, we definitely *can* conclude that the inhibition of necroptosis (regardless whether it is caused by hypomorphic ADAM17 or by loss of RIPK3) does neither ameliorate nor worsen the damage caused by DSS-induced colitis in ADAM17^ex/ex^ mice, in line with the results obtained by Newton *et al.* in WT mice [42]. Therefore, although our own results from DSS-treated WT mice and other studies [14–17] argue that necroptosis does occur in intestinal inflammation, it seems to be dispensable or at least not to significantly contribute to the associated pathology.

Regarding a role of non-necroptotic functions of RIPK3 (such as those suggested by Moriwaki *et al.* [43]) in the hypersensitive response of ADAM17^ex/ex^ mice to DSS-induced colitis, we currently do not know whether hypomorphic ADAM17 impairs only the necroptotic activation (i.e., phosphorylation) of RIPK3 or whether it also interferes with its necroptosis-independent functions. Nevertheless, since our results show no difference between ADAM17^ex/ex^ and ADAM17^ex/ex^/RIPK3^−/−^ mice, even if non-necroptotic functions of RIPK3 are still active in ADAM17^ex/ex^ mice, they are obviously irrelevant for the course of the disease, again consistent with what Newton and coworkers have observed for WT mice [42], and different from the results of Moriwaki and colleagues [43].

Our results furthermore demonstrate that necroptotic signaling is not only compromised in the colon of ADAM17^ex/ex^ mice, but also in the liver. Given that the hypomorphic mutation in these mice affects the entire body, we consider it safe to assume that necroptosis is impaired in every tissue of ADAM17^ex/ex^ mice. Moreover, the data we have obtained in ADAM17^ex/ex^ MEF show that the protection conferred by hypomorphic ADAM17 is not limited to DSS-induced colitis but also encompasses TNF-induced necroptosis. Therefore, it will be interesting to determine whether ADAM17 is similarly important for necroptosis induced by yet other triggers such as TLRs, interferons or the T cell receptor.

Notably, ADAM proteases have already been implicated in the regulation of necroptosis by the work of Cai *et al.* [44]. In their study, the authors showed that ADAMs are activated early in necroptosis downstream of MLKL, and that the subsequent shedding of cell surface proteins promotes necroptosis, cell migration and inflammation. Shedding was mediated by ADAM9 and ADAM10 in human cells or ADAM10 and ADAM17 in murine cells. The authors demonstrated that RIPK3-mediated phosphorylation of MLKL was required for formation of a complex between pMLKL and ADAM10 (or ADAM9, when ADAM10 was knocked down), and speculated that this complex is required for the necroptotic activation of ADAM9 and ADAM10. Double knockdown of ADAM9/ADAM10 (in human cells), double knockout of ADAM10/ADAM17 (in murine cells) or treatment with the specific ADAM metalloproteinase inhibitor GW280264X delayed necroptosis, but only when cells were cultured adherent, not in suspension. The authors suggested that activation of ADAMs plays a key role in promoting necroptosis in adherent cells, probably by disrupting cell adhesion [44].

Regarding the mechanism by which ADAM17 interconnects with the necroptotic signaling machinery, our own data show that hypomorphic ADAM17 impairs RIPK3 and MLKL phosphorylation. This implicates that ADAM17 acts upstream of necrosome formation whereas the data of Cai *et al.* suggest that ADAMs are activated downstream of MLKL. As a point to consider, Cai and colleagues did not address whether ADAM17 also forms a complex with pMLKL during necroptosis. Future experiments will have to clarify whether ADAM17 couples to necroptosis up- or downstream of MLKL, or possibly by two independent mechanisms. Another question to be addressed in more detail in the future is how hypomorphic ADAM17 prevents the phosphorylation and activation of RIPK3 and MLKL. Although immediately evident, a compromised shedding of TNF in ADAM17^ex/ex^ mice is most likely not responsible, since ADAM17^ex/ex^ MEF are still resistant to necroptosis induced by an excess of exogenous TNF. Likewise, our results suggest that the role of ADAM17 in necroptosis does not depend on the regulation of RIPK1 levels. However, in our hands, inhibitors of ADAM proteases did not protect from necroptosis, providing first indications that ADAM17 may control necroptosis by interacting with other proteins rather than by its enzymatic activity.

In summary, our results identify the metalloproteinase ADAM17 as a novel regulator of necroptosis. It will be interesting to further characterize the underlying mechanisms (e.g., by analysis of cells carrying a complete knockout of ADAM17 and their reconstitution with ADAM17 mutants), and also to determine whether ADAM17 has a similar role in controlling the non-necroptotic, pro-inflammatory functions of RIPK3.

## MATERIALS AND METHODS

### Mice

ADAM17^ex/ex^ and RIPK3^−/−^ mice (C57BL/6 background) have been described [13] or were originally obtained from Genentech. Since homozygous ADAM17^ex/ex^ mice show a reduced milk duct development [13] and a reduced efficiency of breeding, both ADAM17^ex/ex^ and ADAM17^ex/ex^/RIPK3^−/−^ animals were generated from crosses of heterozygous ADAM17^WT/ex^ and ADAM17^WT/ex^/RIPK3^−/−^ breeding pairs. The genotypes of all animals were verified by PCR using primers 5’CTTATTATTCTCGTGGTC3’ and 5’TATGTGATAGGTGTAATG3’ for Adam17 and 5’CGCTTTAGAAGCCTTCAGGTTGAC3’, 5’GCCTGC CCATCAGCAACTC3’ and 5’CCAGAGGCCACTTG TGTAGCG3’ for Ripk3. Mice were maintained in a 12-h light-dark cycle under standard conditions and were provided with food and water *ad libitum*. All experiments were performed according to the German Regulations of Animal Welfare approved by the Ministerium für Energiewende, Landwirtschaft, Umwelt, Natur und Digitalisierung, Schleswig-Holstein (Kiel, Germany; V 312-7224.121-20 (18-1/13)).

### DSS treatment

To induce acute colitis, male mice (12-20 weeks old) were administered 1.5% DSS (molecular mass 36,000-50,000 Da; MP Biomedicals, Eschwege, Germany) in the drinking water *ad libitum* for 5 days followed by 5 days of regular drinking water. For induction of chronic colitis, mice were initially provided 0.5% DSS for 5 days, followed by 5 days of regular drinking water. This was repeated for two more cycles with a concentration of 0.75% DSS in each administration phase, resulting in a 30-day experimental period. Control mice had access to untreated water.

### Organ and tissue sample handling

After anesthesia of mice with ketamine/xylazine, whole blood was collected by cardiac puncture in lithium-heparin-coated microvettes and serum was obtained by centrifugation at 10,000 × g for 5 min. Anesthetized mice were sacrificed by cervical dislocation and organs were isolated immediately afterwards. The length of colon and small intestine as well as the weight of caecum, spleen and liver were determined. Subsequently, caecum, spleen and liver were divided in two halves. One half was shock-frozen in liquid nitrogen for further protein analyses, while the other was fixed in 10% w/v formalin for at least 24 h at 4°C. The colon was separated longitudinally into two equal parts. One longitudinal section was rolled up, starting with the distal part, having the distal colon at the very inner layer and the proximal colon at the very outer layer (swiss rolls) and fixed with formalin. The other section was divided in four parts from proximal to distal and shock-frozen in liquid nitrogen for further protein analyses. Formalin-fixed tissue was embedded in paraffin and dissected in 3-4.5 μm sections using the RM2255 microtome (Leica, Wetzlar, Germany) for further histological examination.

### Histology

Histopathological analyses were performed after Mayer’s H&E staining of tissue sections. For immunofluorescence staining, the Tyramide Signal Amplification Cy3 system (Perkin Elmer, Rodgau, Germany) was used, employing antibodies against β-catenin (#8480, Cell Signaling, Leiden, The Netherlands) and myeloperoxidase (ab9535, Abcam, Cambridge, UK). Nuclei were counterstained with Hoechst 33342 (Thermo Fisher, Darmstadt, Germany). Slides were analyzed with a transmitted light microscope (Axio Imager Z1, ZEISS, Oberkochen, Germany) and the AxioVision Rel 4.9 software (ZEISS) or an DMI4000 B microscope (Leica, Wetzlar, Germany). Cell death was assessed by TUNEL staining using the ApopTag Plus Peroxidase *In Situ* Apoptosis Detection Kit (Merck, Darmstadt, Germany) according to the manufacturer’s instructions. Micrographs of β-catenin and myeloperoxidase were adjusted for brightness with Adobe Photoshop CS (Adobe Systems, Mountainview, CA, USA), applying identical settings to all panels of the same set.

### KC ELISA

Murine KC was quantified from serum with the Mouse CXCL1/KC DuoSet® ELISA Development System (BioTechne, Wiesbaden, Germany) according to the manufacturer’s protocol.

### Determination of clinical scores

To calculate the disease activity index, we assessed body weight, stool consistency and rectal bleeding using the haemoccult test (Beckman Coulter, Krefeld-Fischeln, Germany) as follows [24]: no weight loss was scored as 0, weight loss of 1-5% as 1, 6-10% as 2, 11-20% as 3, and more than 20% as 4 points. With regard to stool consistency, 0 points were assigned for well-formed pellets, 2 points for pasty and semiformed stools that did not adhere to the anus, and 4 points for liquid stools that did adhere to the anus. For rectal bleeding, 0 was assigned for negative haemoccult tests, 2 for positive haemoccult reactions, and 4 for rectal bleeding. Histological scoring of H&E-stained colon swiss rolls was performed in a blinded approach by evaluating inflammatory cell infiltration (score 0-3), tissue damage (score 0-5) and ulceration (score 0-3). The combined histological score ranged from 0 (no changes) to 11 (extensive cell infiltration, tissue damage and ulceration).

### Immunoblots

Tissue samples from distal colon and liver were homogenized in RIPA buffer (150 mM NaCl, 1% v/v Triton X-100, 0.5% w/v sodium deoxycholate, 0.1% w/v SDS, 50 mM Tris pH 8.0) containing Complete protease inhibitor (Roche, Mannheim, Germany). Identical amounts of cell protein per lane were resolved by electrophoresis on SDS polyacrylamide gels. After electrophoretic transfer to nitrocellulose, reactive proteins were detected using antibodies specific for phospho S232-RIPK3 (ab195117, Abcam), RIPK3 (PRS2283, Merck), phospho S345-MLKL (ab196436, Abcam), MLKL (orb32399, Biorbyt, Cambridge, UK), cleaved (active) caspase-3 (#9661, Cell Signaling), Bcl-2 (sc-7382, Santa Cruz, Heidelberg, Germany), RIPK1 (610459, BD Biosciences, Heidelberg, Germany), actin (A1978, Merck), and the LumiGLO detection kit (Cell Signaling). Positive control lysates for necroptosis and apoptosis were generated from WT MEF [45] treated for 16 h with 100 ng/ml TNF and 1 μg/ml CHX (Merck) in the presence (necroptosis) or absence (apoptosis) of 20 μM zVAD-fmk. Equal loading as well as efficiency of transfer was routinely verified for all Western blots by reprobing the membranes for actin or alternatively, by Ponceau S staining. Contrast and brightness of digital images was adjusted with Adobe Photoshop CS (Adobe Systems), always applying identical settings to all panels from the same film. Quantifications were performed with ImageJ 1.51.S (Wayne Rasband, National Institutes of Health, Bethesda, MD, USA, http://imagej.nih.gov/ij).

### TNF-induced necroptosis in WT and ADAM17^ex/ex^ MEF

Highly purified human recombinant TNF was provided by BASF Bioresearch (Ludwigshafen, Germany). zVAD-fmk was from Bachem (Bubendorf, Switzerland), GW280264X from Iris Biotech (Marktredwitz, Germany), marimastat from BioTechne and GI254023X and TAPI-1 from Merck. WT and ADAM17^ex/ex^ MEF have been described [13, 46] and were cultivated in DMEM supplemented with 10% v/v calf serum, 50 μg/ml each of streptomycin and penicillin and 50 μM β-mercaptoethanol in a humidified incubator containing 5% w/v CO_2_. Cells were seeded in six-well plates at 5 × 10^5^ cells/well. Following treatment, both detached and adherent cells were collected. For flow cytometric analysis of membrane integrity, the cells were resuspended in PBS/5 mM EDTA containing 2 μg/ml propidium iodide (PI), and the red fluorescence was measured on a FACSCalibur flow cytometer (Becton Dickinson, Heidelberg, Germany). For Western blots, cells were lysed at 4°C in TNE buffer (50 mM Tris pH 8.0, 1% v/v NP40, 2 mM EDTA, Complete protease inhibitor mixture (Roche)), and analyzed by immunoblot as outlined above.

### Statistical analysis

Statistical significance was determined by two-tailed unpaired Student’s *t*-test using GraphPad Prism (GraphPad Software, La Jolla, CA, USA). p values <0.05 were considered statistically significant (^*^, p<0.05; ^**^, p<0.01; ^***^, p<0.001).

## SUPPLEMENTARY MATERIALS FIGURES



## Abbreviations

ADAM: A Disintegrin And Metalloproteinase
ADAM17^ex/ex^: hypomorphic for ADAM17
Bcl-2: B-cell lymphoma 2
CHX: cycloheximide
DAI: disease activity index
DSS: dextran sulfate sodium
EGFR: epidermal growth factor receptor
H&E: hematoxylin and eosin
KC: keratinocyte-derived cytokine
MEF: murine embryonic fibroblasts
MLKL: mixed-lineage kinase domain-like
MPO: myeloperoxidase
PI: propidium iodide
pMLKL: phosphorylated MLKL
pRIPK3: phosphorylated RIPK3
RIPK1: receptor interacting serine-threonine protein kinase 1
RIPK3: receptor interacting serine-threonine protein kinase 3
TACE: TNF-α-converting enzyme
TLRs: Toll-like receptors
TNF: tumor necrosis factor
TUNEL: TdT-mediated dUTP nick end labeling
WT: wildtype
zVAD-fmk: benzyloxycarbonyl-Val-Ala-Asp(OMe)-fluoromethylketone
