# Supplementary material for: The enhanced susceptibility of ADAM-17 hypomorphic mice to DSS-induced colitis is not ameliorated by loss of RIPK3, revealing an unexpected function of ADAM-17 in necroptosis
Source: Oncotarget. 2018 Feb 5;9(16):12941–58. doi: 10.18632/oncotarget.24410 (PMC5849186; doi:10.18632/oncotarget.24410)
Supplement: Supplementary file 1 [file oncotarget-09-12941-s001.pdf]

The enhanced susceptibility of ADAM-17 hypomorphic mice to DSS-induced colitis is not ameliorated by loss of RIPK3, revealing an unexpected function of ADAM-17 in necroptosis

SUPPLEMENTARY MATERIALS

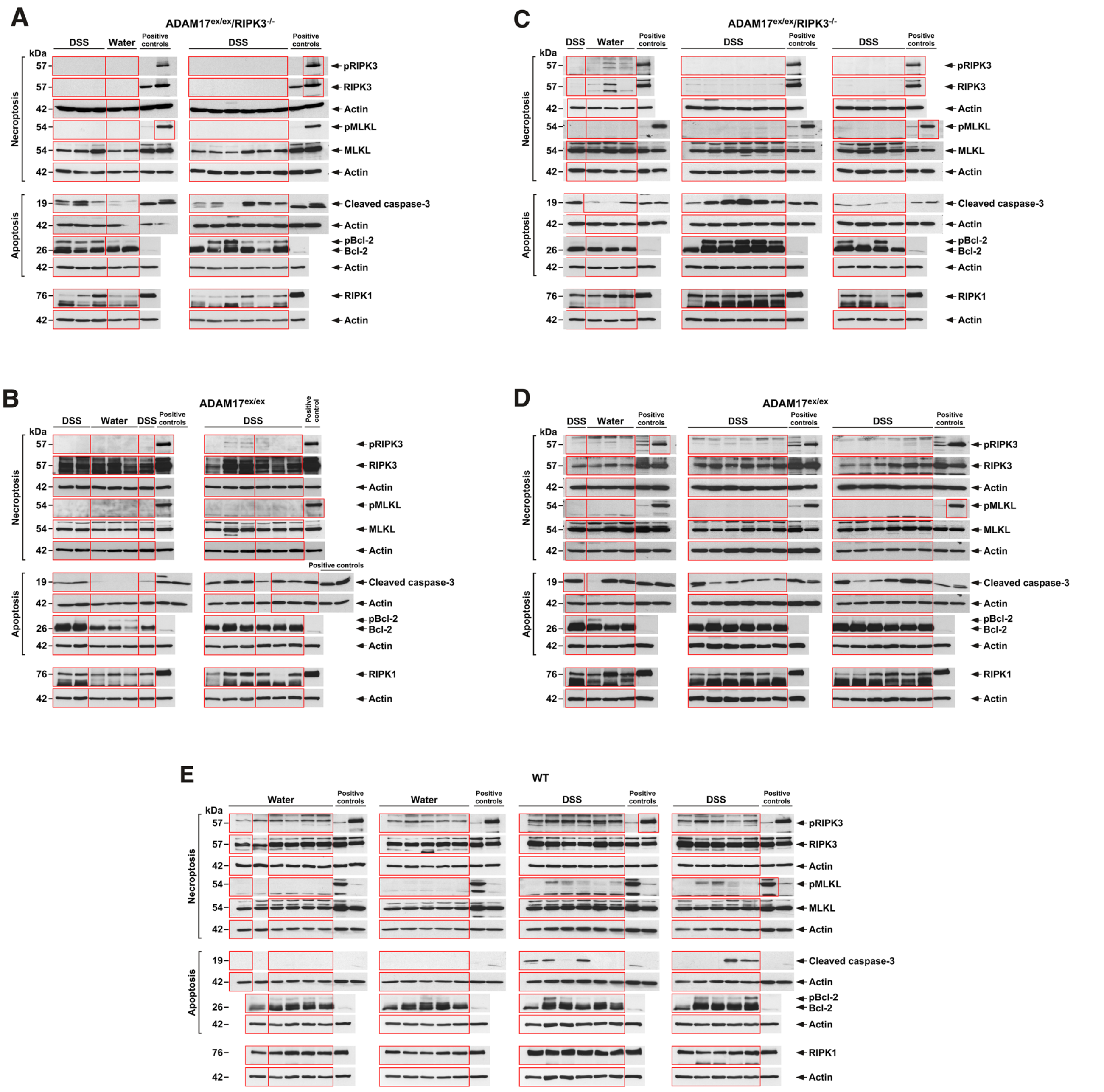

Supplementary Figure 1: (A-E) uncropped Western blot panels for Figure 7. Samples were analyzed on multiple gels with positive control lysates for necroptosis or apoptosis present on each gel. Red boxes mark the areas that were cropped and assembled into Figure 7A-7E.

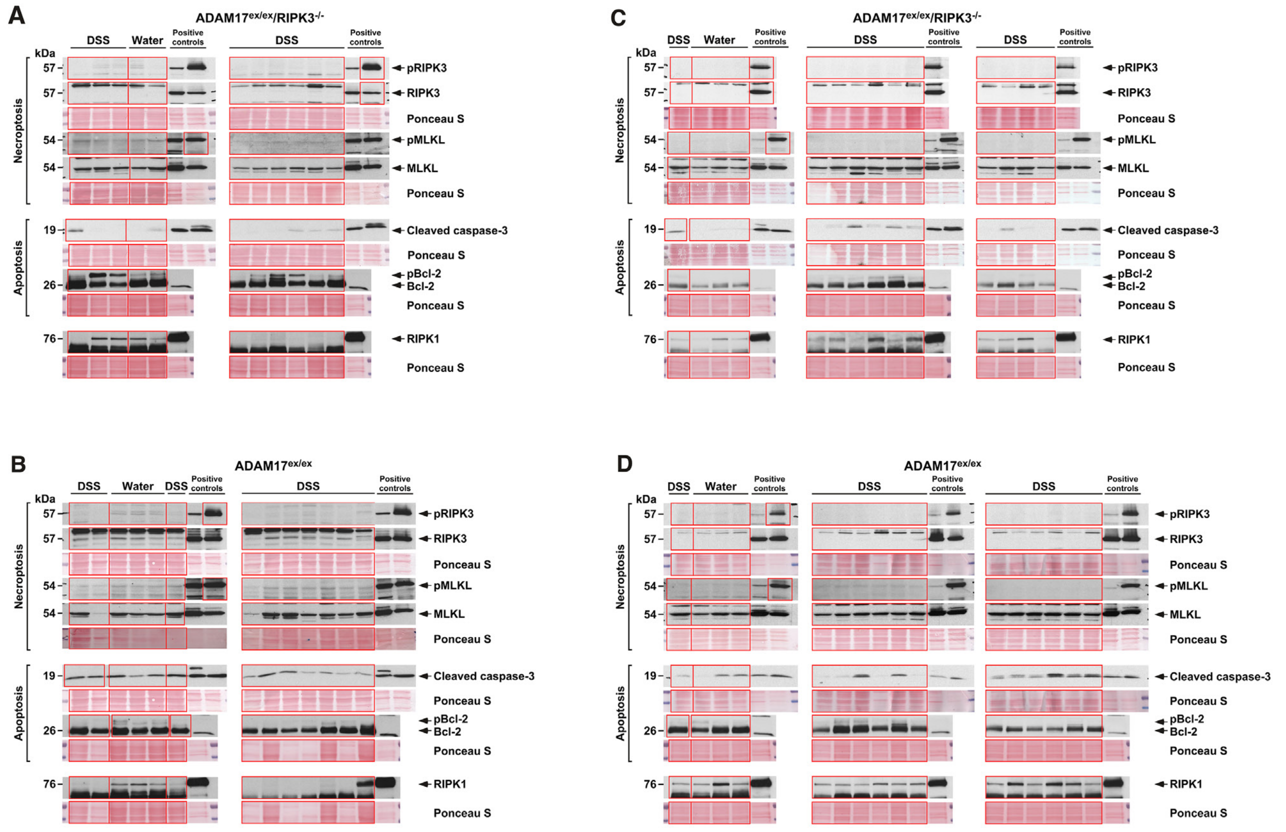

Supplementary Figure 2: (A-D) uncropped Western blot panels for Figure 8. Samples were analyzed on multiple gels with positive control lysates for necroptosis or apoptosis present on each gel. Red boxes mark the areas that were cropped and assembled into Figure 8A-8D.
